# Supplementary material for: Healthcare worker’s emotions, perceived stressors and coping mechanisms during the COVID-19 pandemic
Source: PLoS One. 2021 Jul 9;16(7):e0254252. doi: 10.1371/journal.pone.0254252 (PMC8270181; doi:10.1371/journal.pone.0254252)
Supplement: S1 File — (DOCX) [file pone.0254252.s001.docx]

**S1 File.**

| **Item** | **Variable** | **Category** | **Nursing** | | **Physician** | | **Other** | |
| --- | --- | --- | --- | --- | --- | --- | --- | --- |
|  |  |  | **Count** | **%** | **Count** | **%** | **Count** | **%** |
| 1 | I felt I had to do my job as it is my professional and ethical duty. | Strongly Disagree | 3 | 2.1 | 0 | 0.0 | 0 | 0.0 |
|  |  | Disagree | 2 | 1.4 | 0 | 0.0 | 0 | 0.0 |
|  |  | Neither Agree or Disagree | 8 | 5.6 | 0 | 0.0 | 5 | 6.3 |
|  |  | Agree | 32 | 22.5 | 1 | 4.5 | 16 | 20.3 |
|  |  | Strongly Agree | 97 | 68.3 | 21 | 95.5 | 58 | 73.4 |
| 2 | I felt nervous and scared. | Strongly Disagree | 1 | 0.7 | 3 | 13.6 | 3 | 3.8 |
|  |  | Disagree | 9 | 6.4 | 3 | 13.6 | 9 | 11.3 |
|  |  | Neither Agree or Disagree | 12 | 8.5 | 1 | 4.5 | 11 | 13.8 |
|  |  | Agree | 44 | 31.2 | 11 | 50.0 | 34 | 42.5 |
|  |  | Strongly Agree | 75 | 53.2 | 4 | 18.2 | 23 | 28.7 |
| 3 | I appreciated the special recognition for my job by hospital administration. | Strongly Disagree | 12 | 8.6 | 4 | 18.2 | 7 | 8.9 |
|  |  | Disagree | 17 | 12.1 | 2 | 9.1 | 14 | 17.7 |
|  |  | Neither Agree or Disagree | 35 | 25.0 | 8 | 36.4 | 18 | 22.8 |
|  |  | Agree | 48 | 34.3 | 3 | 13.6 | 28 | 35.4 |
|  |  | Strongly Agree | 28 | 20.0 | 5 | 22.7 | 12 | 15.2 |
| 4 | I thought of quitting my job. | Strongly Disagree | 58 | 41.4 | 12 | 54.5 | 36 | 47.4 |
|  |  | Disagree | 34 | 24.3 | 7 | 31.8 | 20 | 26.3 |
|  |  | Neither Agree or Disagree | 15 | 10.7 | 0 | 0.0 | 7 | 9.2 |
|  |  | Agree | 23 | 16.4 | 2 | 9.1 | 7 | 9.2 |
|  |  | Strongly Agree | 10 | 7.1 | 1 | 4.5 | 6 | 7.9 |
| 5 | I would quit my job if a COVID-19 outbreak recurred. | Strongly Disagree | 67 | 47.9 | 14 | 63.6 | 39 | 50.0 |
|  |  | Disagree | 37 | 26.4 | 6 | 27.3 | 24 | 30.8 |
|  |  | Neither Agree or Disagree | 28 | 20.0 | 2 | 9.1 | 9 | 11.5 |
|  |  | Agree | 4 | 2.9 | 0 | 0.0 | 5 | 6.4 |
|  |  | Strongly Agree | 4 | 2.9 | 0 | 0.0 | 1 | 1.3 |
| 6 | I thought of calling in sick. | Strongly Disagree | 61 | 44.2 | 17 | 77.3 | 36 | 45.6 |
|  |  | Disagree | 36 | 26.1 | 4 | 18.2 | 24 | 30.4 |
|  |  | Neither Agree or Disagree | 10 | 7.2 | 1 | 4.5 | 10 | 12.7 |
|  |  | Agree | 22 | 15.9 | 0 | 0.0 | 5 | 6.3 |
|  |  | Strongly Agree | 9 | 6.5 | 0 | 0.0 | 4 | 5.1 |
| 7 | I called in sick at least once during the outbreak. | Yes | 21 | 14.8 | 3 | 13.6 | 10 | 12.8 |
|  |  | No | 121 | 85.2 | 19 | 86.4 | 68 | 87.2 |
| 8 | It stressed me to see my colleagues getting sick. | Strongly Disagree | 4 | 3.1 | 1 | 5.0 | 2 | 2.7 |
|  |  | Disagree | 8 | 6.1 | 0 | 0.0 | 2 | 2.7 |
|  |  | Neither Agree or Disagree | 21 | 16.0 | 1 | 5.0 | 8 | 10.7 |
|  |  | Agree | 43 | 32.8 | 9 | 45.0 | 38 | 50.7 |
|  |  | Strongly Agree | 55 | 42.0 | 9 | 45.0 | 25 | 33.3 |
| 9 | It stressed me to think that I could transmit COVID-19 to my family and friends. | Strongly Disagree | 1 | 0.7 | 1 | 4.5 | 0 | 0.0 |
|  |  | Disagree | 2 | 1.4 | 0 | 0.0 | 3 | 3.8 |
|  |  | Neither Agree or Disagree | 2 | 1.4 | 2 | 9.1 | 3 | 3.8 |
|  |  | Agree | 30 | 21.1 | 5 | 22.7 | 23 | 28.7 |
|  |  | Strongly Agree | 107 | 75.4 | 14 | 63.6 | 51 | 63.7 |
| 10 | It stressed me to see patients with COVID-19 dying in front of me. | Strongly Disagree | 2 | 1.6 | 1 | 5.0 | 0 | 0.0 |
|  |  | Disagree | 2 | 1.6 | 0 | 0.0 | 3 | 4.6 |
|  |  | Neither Agree or Disagree | 6 | 4.9 | 1 | 5.0 | 6 | 9.2 |
|  |  | Agree | 44 | 35.8 | 9 | 45.0 | 23 | 35.4 |
|  |  | Strongly Agree | 69 | 56.1 | 9 | 45.0 | 33 | 50.8 |
| 11 | It was stressful not knowing when the COVID-19 pandemic will be under control. | Strongly Disagree | 0 | 0.0 | 1 | 4.5 | 1 | 1.3 |
|  |  | Disagree | 4 | 2.8 | 0 | 0.0 | 1 | 1.3 |
|  |  | Neither Agree or Disagree | 3 | 2.1 | 3 | 13.6 | 4 | 5.1 |
|  |  | Agree | 33 | 23.1 | 4 | 18.2 | 28 | 35.4 |
|  |  | Strongly Agree | 103 | 72.0 | 14 | 63.6 | 45 | 57.0 |
| 12 | I was stressed because I was emotionally exhausted. | Strongly Disagree | 1 | 0.7 | 4 | 18.2 | 2 | 2.5 |
|  |  | Disagree | 3 | 2.1 | 3 | 13.6 | 9 | 11.3 |
|  |  | Neither Agree or Disagree | 18 | 12.7 | 1 | 4.5 | 6 | 7.5 |
|  |  | Agree | 42 | 29.6 | 7 | 31.8 | 26 | 32.5 |
|  |  | Strongly Agree | 78 | 54.9 | 7 | 31.8 | 37 | 46.3 |
| 13 | I was stressed because I was physically stressed /fatigued. | Strongly Disagree | 3 | 2.1 | 3 | 13.6 | 3 | 3.8 |
|  |  | Disagree | 8 | 5.7 | 3 | 13.6 | 7 | 8.9 |
|  |  | Neither Agree or Disagree | 16 | 11.3 | 4 | 18.2 | 12 | 15.2 |
|  |  | Agree | 46 | 32.6 | 8 | 36.4 | 26 | 32.9 |
|  |  | Strongly Agree | 68 | 48.2 | 4 | 18.2 | 31 | 39.2 |
| 14 | I was stressed because I experienced conflict between my duty and my own safety. | Strongly Disagree | 10 | 7.0 | 3 | 13.6 | 4 | 5.0 |
|  |  | Disagree | 13 | 9.2 | 5 | 22.7 | 14 | 17.5 |
|  |  | Neither Agree or Disagree | 21 | 14.8 | 2 | 9.1 | 12 | 15.0 |
|  |  | Agree | 33 | 23.2 | 7 | 31.8 | 23 | 28.7 |
|  |  | Strongly Agree | 65 | 45.8 | 5 | 22.7 | 27 | 33.8 |
| 15 | I was stressed because I felt there were not adequate protective measures. | Strongly Disagree | 5 | 3.5 | 4 | 18.2 | 4 | 5.1 |
|  |  | Disagree | 9 | 6.3 | 3 | 13.6 | 18 | 23.1 |
|  |  | Neither Agree or Disagree | 13 | 9.1 | 2 | 9.1 | 10 | 12.8 |
|  |  | Agree | 38 | 26.6 | 7 | 31.8 | 19 | 24.4 |
|  |  | Strongly Agree | 78 | 54.5 | 6 | 27.3 | 27 | 34.6 |
| 16 | I was stressed because there was shortage of staff at times. | Strongly Disagree | 4 | 2.9 | 4 | 18.2 | 3 | 3.9 |
|  |  | Disagree | 12 | 8.8 | 6 | 27.3 | 8 | 10.5 |
|  |  | Neither Agree or Disagree | 14 | 10.2 | 1 | 4.5 | 10 | 13.2 |
|  |  | Agree | 36 | 26.3 | 7 | 31.8 | 26 | 34.2 |
|  |  | Strongly Agree | 71 | 51.8 | 4 | 18.2 | 29 | 38.2 |
| 17 | My stress reduced when I saw improvement in patient’s condition. | Strongly Disagree | 1 | 0.7 | 0 | 0.0 | 1 | 1.4 |
|  |  | Disagree | 10 | 7.3 | 1 | 4.8 | 2 | 2.8 |
|  |  | Neither Agree or Disagree | 27 | 19.7 | 6 | 28.6 | 13 | 18.1 |
|  |  | Agree | 67 | 48.9 | 11 | 52.4 | 44 | 61.1 |
|  |  | Strongly Agree | 32 | 23.4 | 3 | 14.3 | 12 | 16.7 |
| 18 | My stress reduced because of the protective equipment provided to me by the hospital. | Strongly Disagree | 20 | 14.2 | 1 | 4.5 | 6 | 7.5 |
|  |  | Disagree | 35 | 24.8 | 3 | 13.6 | 15 | 18.8 |
|  |  | Neither Agree or Disagree | 30 | 21.3 | 4 | 18.2 | 18 | 22.5 |
|  |  | Agree | 43 | 30.5 | 9 | 40.9 | 29 | 36.3 |
|  |  | Strongly Agree | 13 | 9.2 | 5 | 22.7 | 12 | 15.0 |
| 19 | My stress reduced because all healthcare professionals were working together on the front line. | Strongly Disagree | 0 | 0.0 | 0 | 0.0 | 2 | 2.5 |
|  |  | Disagree | 12 | 8.4 | 1 | 4.5 | 4 | 5.1 |
|  |  | Neither Agree or Disagree | 17 | 11.9 | 4 | 18.2 | 13 | 16.5 |
|  |  | Agree | 53 | 37.1 | 5 | 22.7 | 31 | 39.2 |
|  |  | Strongly Agree | 61 | 42.7 | 12 | 54.5 | 29 | 36.7 |
| 20 | My stress reduced because of my confidence in the hospital staff in case I got sick from COVID-19. | Strongly Disagree | 6 | 4.3 | 1 | 4.8 | 3 | 3.8 |
|  |  | Disagree | 28 | 20.3 | 2 | 9.5 | 15 | 18.8 |
|  |  | Neither Agree or Disagree | 53 | 38.4 | 6 | 28.6 | 29 | 36.3 |
|  |  | Agree | 32 | 23.2 | 6 | 28.6 | 21 | 26.3 |
|  |  | Strongly Agree | 19 | 13.8 | 6 | 28.6 | 12 | 15.0 |
| 21 | My stress reduced when I shared jokes or humor with colleagues. | Strongly Disagree | 1 | 0.7 | 0 | 0.0 | 1 | 1.4 |
|  |  | Disagree | 6 | 4.3 | 1 | 4.8 | 2 | 2.7 |
|  |  | Neither Agree or Disagree | 18 | 13.0 | 4 | 19.0 | 12 | 16.2 |
|  |  | Agree | 63 | 45.7 | 7 | 33.3 | 36 | 48.6 |
|  |  | Strongly Agree | 50 | 36.2 | 9 | 42.9 | 23 | 31.1 |
| 22 | My stress reduced when I got free meals from the hospital/ community. | Strongly Disagree | 2 | 1.4 | 2 | 9.5 | 2 | 2.6 |
|  |  | Disagree | 10 | 7.2 | 2 | 9.5 | 6 | 7.9 |
|  |  | Neither Agree or Disagree | 25 | 18.0 | 9 | 42.9 | 20 | 26.3 |
|  |  | Agree | 55 | 39.6 | 3 | 14.3 | 26 | 34.2 |
|  |  | Strongly Agree | 47 | 33.8 | 5 | 23.8 | 22 | 28.9 |
| 23 | Getting Daily COVID updates from the hospital leadership helped reduce my stress. | Strongly Disagree | 3 | 2.1 | 2 | 9.1 | 1 | 1.3 |
|  |  | Disagree | 7 | 4.9 | 3 | 13.6 | 2 | 2.6 |
|  |  | Neither Agree or Disagree | 28 | 19.7 | 8 | 36.4 | 18 | 23.1 |
|  |  | Agree | 61 | 43.0 | 6 | 27.3 | 42 | 53.8 |
|  |  | Strongly Agree | 43 | 30.3 | 3 | 13.6 | 15 | 19.2 |
| 24 | Meeting with members of the Army to talk about the stress I was experiencing helped to reduce my stress. | Strongly Disagree | 2 | 2.0 | 1 | 6.7 | 5 | 8.6 |
|  |  | Disagree | 17 | 17.3 | 5 | 33.3 | 4 | 6.9 |
|  |  | Neither Agree or Disagree | 36 | 36.7 | 5 | 33.3 | 27 | 46.6 |
|  |  | Agree | 33 | 33.7 | 2 | 13.3 | 14 | 24.1 |
|  |  | Strongly Agree | 10 | 10.2 | 2 | 13.3 | 8 | 13.8 |
| 25 | I followed strict personal protective measures (e.g. mask, face shield, gown, hand washing etc) as a personal coping strategy. | Strongly Disagree | 0 | 0.0 | 1 | 4.5 | 0 | 0.0 |
|  |  | Disagree | 0 | 0.0 | 2 | 9.1 | 2 | 2.5 |
|  |  | Neither Agree or Disagree | 7 | 4.9 | 0 | 0.0 | 6 | 7.4 |
|  |  | Agree | 51 | 35.7 | 7 | 31.8 | 26 | 32.1 |
|  |  | Strongly Agree | 85 | 59.4 | 12 | 54.5 | 47 | 58.0 |
| 26 | I kept separate clothes for work to minimize transmission as a personal coping strategy. | Strongly Disagree | 1 | 0.7 | 1 | 4.5 | 0 | 0.0 |
|  |  | Disagree | 4 | 2.8 | 1 | 4.5 | 3 | 3.7 |
|  |  | Neither Agree or Disagree | 5 | 3.5 | 2 | 9.1 | 7 | 8.6 |
|  |  | Agree | 44 | 31.0 | 5 | 22.7 | 28 | 34.6 |
|  |  | Strongly Agree | 88 | 62.0 | 13 | 59.1 | 43 | 53.1 |
| 27 | I did relaxation activities, e.g. involved in prayers, exercise etc. as a personal coping strategy. | Strongly Disagree | 3 | 2.1 | 3 | 14.3 | 2 | 2.5 |
|  |  | Disagree | 13 | 9.1 | 2 | 9.5 | 5 | 6.2 |
|  |  | Neither Agree or Disagree | 26 | 18.2 | 3 | 14.3 | 14 | 17.3 |
|  |  | Agree | 55 | 35.5 | 4 | 19.0 | 35 | 43.2 |
|  |  | Strongly Agree | 46 | 32.2 | 9 | 42.9 | 25 | 30.9 |
| 28 | I chatted with family and friends to relieve stress and obtain support as a personal coping strategy. | Strongly Disagree | 8 | 5.6 | 3 | 13.6 | 1 | 1.2 |
|  |  | Disagree | 12 | 8.4 | 1 | 4.5 | 2 | 2.5 |
|  |  | Neither Agree or Disagree | 17 | 11.9 | 2 | 9.1 | 7 | 8.6 |
|  |  | Agree | 52 | 36.4 | 8 | 36.4 | 41 | 50.6 |
|  |  | Strongly Agree | 54 | 37.8 | 8 | 36.4 | 30 | 37.0 |
| 29 | I talked to myself and motivated myself to face the COVID-19 pandemic with positive attitude as a personal coping strategy. | Strongly Disagree | 3 | 2.1 | 3 | 13.6 | 2 | 2.5 |
|  |  | Disagree | 8 | 5.6 | 4 | 18.2 | 6 | 7.5 |
|  |  | Neither Agree or Disagree | 19 | 13.4 | 6 | 27.3 | 16 | 20.0 |
|  |  | Agree | 65 | 45.8 | 5 | 22.7 | 35 | 43.8 |
|  |  | Strongly Agree | 47 | 33.1 | 4 | 18.2 | 21 | 26.3 |
| 30 | I got help from family physicians or other doctors/ therapists to reduce my stress and get reassurance. | Strongly Disagree | 23 | 16.1 | 9 | 40.9 | 10 | 12.5 |
|  |  | Disagree | 42 | 29.4 | 6 | 27.3 | 30 | 37.5 |
|  |  | Neither Agree or Disagree | 36 | 25.2 | 4 | 18.2 | 23 | 28.7 |
|  |  | Agree | 20 | 14.0 | 2 | 9.1 | 12 | 15.0 |
|  |  | Strongly Agree | 22 | 15.4 | 1 | 4.5 | 5 | 6.3 |
| 31 | I avoided media news about COVID-19 and related fatalities as a coping strategy. | Strongly Disagree | 13 | 9.3 | 6 | 27.3 | 6 | 7.4 |
|  |  | Disagree | 40 | 28.6 | 7 | 31.8 | 17 | 21.0 |
|  |  | Neither Agree or Disagree | 27 | 19.3 | 2 | 9.1 | 24 | 29.6 |
|  |  | Agree | 32 | 22.9 | 2 | 9.1 | 19 | 23.5 |
|  |  | Strongly Agree | 28 | 20.0 | 5 | 22.7 | 15 | 18.5 |
| 32 | I vented emotions by crying, screaming etc. | Strongly Disagree | 16 | 11.2 | 11 | 50.0 | 18 | 22.2 |
|  |  | Disagree | 31 | 21.7 | 4 | 18.2 | 19 | 23.5 |
|  |  | Neither Agree or Disagree | 25 | 17.5 | 3 | 13.6 | 15 | 18.5 |
|  |  | Agree | 45 | 31.5 | 2 | 9.1 | 18 | 22.2 |
|  |  | Strongly Agree | 26 | 18.2 | 2 | 9.1 | 11 | 13.6 |
| 33 | Adequate personal protective supplies provided by the hospital could promote my willingness to participate in any future epidemic/pandemics. | Strongly Disagree | 4 | 2.8 | 0 | 0.0 | 2 | 2.5 |
|  |  | Disagree | 7 | 4.9 | 0 | 0.0 | 1 | 1.2 |
|  |  | Neither Agree or Disagree | 9 | 6.3 | 1 | 4.5 | 7 | 8.6 |
|  |  | Agree | 43 | 30.1 | 6 | 27.3 | 30 | 37.0 |
|  |  | Strongly Agree | 80 | 55.9 | 15 | 68.2 | 41 | 50.6 |
| 34 | Available cure or vaccine for the disease could promote my willingness to participate in any future epidemic/pandemics. | Strongly Disagree | 4 | 2.8 | 0 | 0.0 | 2 | 2.5 |
|  |  | Disagree | 8 | 5.6 | 0 | 0.0 | 5 | 6.2 |
|  |  | Neither Agree or Disagree | 26 | 18.3 | 1 | 4.5 | 23 | 28.4 |
|  |  | Agree | 43 | 30.3 | 8 | 36.4 | 21 | 25.9 |
|  |  | Strongly Agree | 61 | 43.0 | 13 | 59.1 | 30 | 37.0 |
| 35 | Financial recognition of efforts could promote my willingness to participate in any future epidemic/pandemics. | Strongly Disagree | 3 | 2.1 | 1 | 4.5 | 1 | 1.3 |
|  |  | Disagree | 5 | 3.5 | 1 | 4.5 | 1 | 1.3 |
|  |  | Neither Agree or Disagree | 12 | 8.4 | 3 | 13.6 | 7 | 8.8 |
|  |  | Agree | 36 | 25.2 | 7 | 31.8 | 17 | 21.3 |
|  |  | Strongly Agree | 87 | 60.8 | 10 | 45.5 | 54 | 67.5 |
| 36 | Recognition from management and supervisors for the extra efforts could promote my willingness to participate in any future epidemic/pandemics. | Strongly Disagree | 4 | 2.8 | 1 | 4.5 | 0 | 0.0 |
|  |  | Disagree | 7 | 4.9 | 1 | 4.5 | 1 | 1.2 |
|  |  | Neither Agree or Disagree | 22 | 15.4 | 5 | 22.7 | 15 | 18.5 |
|  |  | Agree | 47 | 32.9 | 5 | 22.7 | 20 | 24.7 |
|  |  | Strongly Agree | 63 | 44.1 | 10 | 45.5 | 45 | 55.6 |
| 37 | Psychiatric help and therapy made available in work place to help reduce stress and anxiety could promote my willingness to participate in any future epidemic/pandemics. | Strongly Disagree | 6 | 4.2 | 3 | 13.6 | 2 | 2.5 |
|  |  | Disagree | 11 | 7.7 | 3 | 13.6 | 6 | 7.5 |
|  |  | Neither Agree or Disagree | 48 | 33.6 | 11 | 50.0 | 28 | 35.0 |
|  |  | Agree | 39 | 27.3 | 1 | 4.5 | 22 | 27.5 |
|  |  | Strongly Agree | 39 | 27.3 | 4 | 18.2 | 22 | 27.5 |
| 38 | Reduced working hours during outbreaks could promote my willingness to participate in any future epidemic/pandemics. | Strongly Disagree | 4 | 2.8 | 3 | 13.6 | 3 | 3.9 |
|  |  | Disagree | 15 | 10.5 | 3 | 13.6 | 7 | 8.6 |
|  |  | Neither Agree or Disagree | 49 | 34.3 | 6 | 27.3 | 31 | 38.3 |
|  |  | Agree | 36 | 25.2 | 5 | 22.7 | 19 | 23.5 |
|  |  | Strongly Agree | 39 | 27.3 | 5 | 22.7 | 21 | 25.9 |
