# Supplementary material for: Healthcare worker’s emotions, perceived stressors and coping mechanisms during the COVID-19 pandemic
Source: PLoS One. 2021 Jul 9;16(7):e0254252. doi: 10.1371/journal.pone.0254252 (PMC8270181; doi:10.1371/journal.pone.0254252)
Supplement: Attachment — Submitted filename: Response to Reviewers.docx [file pone.0254252.s003.docx]

Dear Ismaeel Yunusa, PharmD, PhD,

Thank you for the opportunity to revise and resubmit our manuscript (PONE-D-21-11310) entitled “Healthcare Worker’s Emotions, Perceived Stressors and Coping Mechanisms During the COVID-19 Pandemic.” Below are the reviewer comments with our responses and corresponding revisions. The changes that were made are displayed in the attached revised versions of the manuscript (track changes and clean versions) uploaded within this resubmission.

Thank you for your time and we look forward to hearing from you,

Suzanne Rose, MS, PhD, CCRC, FACRP

| Comment | Response |
| --- | --- |
| Reviewer #1 | |
| Is the manuscript technically sound, and do the data support the conclusions?  Reviewer #1: Yes  Reviewer #2: Partly  The manuscript must describe a technically sound piece of scientific research with data that supports the conclusions. Experiments must have been conducted rigorously, with appropriate controls, replication, and sample sizes. The conclusions must be drawn appropriately based on the data presented. | We appreciate your feedback. To clarify, this study did not use controls due to the study design being cross-sectional in nature. This has been added in the methods section. Please see line 106.  We additionally understand that our study’s sample is comprised of mostly female healthcare workers. While this can be seen as a limitation, it was recently cited that the number of full-time, year-round workers in healthcare occupations has almost doubled since 2000, increasing from 5 million to 9 million workers, with women accounting for three-quarters of these personnel.(1,2) |
| Has the statistical analysis been performed appropriately and rigorously?  Reviewer #1: No  Reviewer #2: No | Please see the below responses to reviewers regarding their specific statistical analyses feedback and our crafted responses to address and incorporate this feedback |
| Have the authors made all data underlying the findings in their manuscript fully available?  The PLOS Data policy requires authors to make all data underlying the findings described in their manuscript fully available without restriction, with rare exception (please refer to the Data Availability Statement in the manuscript PDF file). The data should be provided as part of the manuscript or its supporting information, or deposited to a public repository. For example, in addition to summary statistics, the data points behind means, medians and variance measures should be available. If there are restrictions on publicly sharing data—e.g. participant privacy or use of data from a third party—those must be specified. | In order for readers to understand the study fully, we have uploaded our de-identified dataset of the survey responses into a data repository. This can be found at DOI: doi.org/10.3886/E142222V1  We have also included the database for your review and possible publication as a supplemental file. This file contains the raw data that was collected; readers may download the file from the data repository or via downloading this manuscript’s Supporting Information database file included in this revised submission |
| Is the manuscript presented in an intelligible fashion and written in standard English?  Reviewer #1: Yes  Reviewer #2: Yes  PLOS ONE does not copy edit accepted manuscripts, so the language in submitted articles must be clear, correct, and unambiguous. Any typographical or grammatical errors should be corrected at revision, so please note any specific errors here. | Thank you for your comment |
| Reviewer Comments to Authors | |
| ***Reviewer #1:*** | |
| This is a very timely manuscript exploring the perceived stressors and coping mechanisms as experienced by HCWs, who were in contact with COVID19 patients during the first wave of the COVID-19 pandemic at Stamford hospital It is a very detailed questionnaire based cross sectional survey. Although I agree with the statistical methods employed, it is quite confusing to interpret the questions answered by the study participants. It would be more clear and easy to follow if the *questions and its responses were answered as sliding scale with percentages of responses for all the 5 likert scale responses to get a sense of how each question was answered across the whole group or within subgroups specified by the authors* | In order for readers to understand the study fully, we have uploaded our de-identified dataset of the survey responses into a data repository. The data can be found at DOI: doi.org/10.3886/E142222V1  We have included additional data for your review and possible publication as a supplemental file. This file contains the Likert score results for each individual question and response item with percentages of responses for all the 5 Likert scale responses across the specific subgroups specified by the authors . The raw data can also be utilized to calculate the percentages of responses for all the 5 Likert scale responses across the whole group.  Readers may also download the raw data file from the data repository or via downloading this manuscript’s Supporting Information database file included in this revised submission |
| ***Reviewer #2:*** | |
| This is an interesting and relevant study. The authors aimed to address a timely research and clinical gap of knowledge among Healthcare workers (HCWs). The paper is generally well written and structured.  However, in my opinion the paper has a major limitation in regards to the data analyses.  *The data were not tested for statistical assumptions for ANOVA such as normality test, etc., prior to the analysis. So, it is not possible to know if the ANOVA used in the analysis was appropriate or not. Using the ANOVA test inappropriately can invalidate the results and the study's conclusion. I will suggest the authors to run the analysis again (to test the assumptions) to see if the ANOVA or Kruskall Wallis is most suitable for this data.* | Thank you for your feedback.  Each reviewer had distinctly different approaches to the analysis. In looking at both suggestions, we have provided the de-identified dataset of the survey responses into a data repository. This can be found at DOI: doi.org/10.3886/E142222V1. It is hoped that any reader can then utilize and further analyze the data or subsets of the data.  We have further responded to this concern in the box below where weighing of the study sample is suggested. |
| The response is very low, and the authors did not mention how the rate was calculated in the methods section. the authors failed to weigh the study samples, the literature suggests "weighing" a study sample when a response rate is around 20% (Jack 2008).  (<https://www.ncbi.nlm.nih.gov/> pmc/articles/PMC2384218/#:~:text=Response rates approximating 60% for,of ≥ 80% is expected.) The authors should state the efforts made to improve the response rate. | Although the response rate was indeed low, we sent this survey out to all of our frontline workers. This being said, there are more than 1,000 included individuals within this target population. Employees may have been on vacation or working remotely so they did not see the study announcement. The study was open for a period of one month in order to capture as many responses as possible while also being sensitive to the timely nature of the data collection as a second COVID-19 wave of the pandemic was anticipated to affect our health system in the Fall/Winter months.  We also appreciate the suggestions regarding weighing of the responses when the response rate is around 20%. This approach while viable provided us with some concerns addressed below:   - Gamma (skew) statistics could be used with an absolute value of \|2\| and values within the range would be analyzed with ANOVA and observed means (SDs) reported. - Values outside of the \|2\| range would then be weighted and the weighted values reported. While this is a valid technique it would require reporting two large tables instead of the count and percent table below. - Within the two tables one table would have observed means and SDs and the other would present weighted means and SDs. This would inhibit the ability to directly compare across items in the questionnaire. - Median analysis could be reported instead of weighting as the reviewer suggests but that would then require two larger tables again. One with Medians and one with Means and SDs. - When we utilized this method, we were presented interesting examples. One such included a medians analysis with Kruskal-Wallis test demonstrated a significant difference between ‘roles’ (p=0.027), however the medians for all three groups is 5.0 (Strongly Agree).   Finally, due to the complexity of the analysis, the increased number of tables, and the lack of the ability to directly compare items across the different analysis methods we decided that the statistical approach from Reviewer 1 would provide the reader with the ability to directly compare items across all items and within each item. |
| In the methods section, it was not clear how the data was collected? was it face-to-face, via email etc. | Please see line 116 stating the data was collected electronically to provide clarification on data collection methods. |
| No sample size calculation. | This survey utilized convenience sample of everyone who answered. This has been incorporated into the methods section in line 120. |
| Over 80% of the sample were females. This should be mentioned in the limitation section. | Our sample is comprised of mostly female healthcare workers. Although the fact that our sample comprised of mostly female healthcare workers, it was recently cited that the number of full-time, year-round workers in healthcare occupations has almost doubled since 2000, increasing from 5 million to 9 million workers, with women accounting for three-quarters of these personnel.(1,2) Although this is true, we decided to include this information within the limitations section of our manuscript. Please see lines 310-315 for the revisions made. |
| I will suggest the first paragraph of the discussion section to explain the key findings and how it answer the research questions. | Thank you for your feedback. We have condensed the discussion section to explain the key findings of our research |
| The conclusion section should be brief and focus on the main study findings. | Thank you for your comments. We condensed the conclusion section to primarily focus on the study findings and next steps for future research and initiatives |
| **Additional Editor Comments:** | |
| Please submit a revised version along with a STROBE (https://www.equator-network.org/reporting-guidelines/strobe/) checklist for reporting observational cross-sectional studies. | Please see the attached STROBE checklist for reporting observational cross-sectional studies |
| Also, concise your Introduction to 3-4 paragraphs. This will encourage busy physicians to read your interesting study. | We appreciate this important and relevant feedback. We have condensed the introduction significantly. Please see lines 67-104 for the revisions made |
| ***Journal Requirements:*** | |
| Please ensure that your manuscript meets PLOS ONE's style requirements, including those for file naming. | All headers and sub-headers within the manuscript are in the proper formatting, and when submitting we will ensure the proper file names for each document meet PLOS ONE’s style requirements |
| We note that you have indicated that data from this study are available upon request. PLOS only allows data to be available upon request if there are legal or ethical restrictions on sharing data publicly. | In order for readers to understand the study fully, we have uploaded our de-identified dataset of the survey responses into a data repository. DOI: doi.org/10.3886/E142222V1  We have also included the database for your review and possible publication as a supplemental file. This file contains the raw Likert score results for each individual question and response item. Readers may download the file from the data repository or via downloading this manuscript’s Supporting Information database file included in this revised submission |
| If there are ethical or legal restrictions on sharing a de-identified data set, please explain them in detail (e.g., data contain potentially sensitive information, data are owned by a third-party organization, etc.) and who has imposed them (e.g., an ethics committee). Please also provide contact information for a data access committee, ethics committee, or other institutional body to which data requests may be sent. |  |
| If there are no restrictions, please upload the minimal anonymized data set necessary to replicate your study findings as either Supporting Information files or to a stable, public repository and provide us with the relevant URLs, DOIs, or accession numbers. |  |
| We note that you have included the phrase “**data not shown**” in your manuscript. Unfortunately, this does not meet our data sharing requirements. PLOS does not permit references to inaccessible data. We require that authors provide all relevant data within the paper, Supporting Information files, or in an acceptable, public repository. Please add a citation to support this phrase or upload the data that corresponds with these findings to a stable repository (such as Figshare or Dryad) and provide and URLs, DOIs, or accession numbers that may be used to access these data. Or, if the data are not a core part of the research being presented in your study, we ask that you remove the phrase that refers to these data. | This specific data referenced is incorporated in the Supporting Information database file and the revisions made to reflect this change. This file contains the raw Likert score results for each individual question and response item. Readers may download the file from the data repository or via downloading the manuscript Supporting Information database file. DOI: doi.org/10.3886/E142222V1 |

References:

1.	US Census Bureau. Your Health Care Is in Women’s Hands [Internet]. The United States Census Bureau. [cited 2021 Jun 9]. Available from: https://www.census.gov/library/stories/2019/08/your-health-care-in-womens-hands.html

2.	United Nations. COVID-19 and gender: What do we know; what do we need to know? [Internet]. UN Women Data Hub. 2020 [cited 2021 Jun 11]. Available from: https://data.unwomen.org/features/covid-19-and-gender-what-do-we-know-what-do-we-need-know
